# Benfotiamine Counteracts Smoking-Induced Vascular Dysfunction in Healthy Smokers

**DOI:** 10.1155/2012/968761

**Published:** 2012-10-03

**Authors:** Alin Stirban, Simona Nandrean, Stanley Kirana, Christian Götting, Ioan Andrei Veresiu, Diethelm Tschoepe

**Affiliations:** ^1^Diabetes Center, Heart and Diabetes Center NRW Bad Oeynhausen, Ruhr University of Bochum, Georgstrasse 11, 32545 Bad Oeynhausen, Germany; ^2^Profil Institute for Metabolic Research, Hellersbergstrasse 9, 41460 Neuss, Germany; ^3^Institute for Laboratory and Transfusion Medicine, Heart and Diabetes Center NRW, Bad Oeynhausen, Ruhr University of Bochum, Georgstrasse 11, 32545 Bad Oeynhausen, Germany; ^4^Center for Diabetes, Nutrition and Metabolic Diseases, “Iuliu Hatieganu” University, Str. Clinicilor nr. 2, 400006 Cluj-Napoca, Romania

## Abstract

*Background*. Smoking induces endothelial dysfunction (ED) mainly by exacerbating oxidative stress (OS) and inflammation. Benfotiamine, a thiamine prodrug with high bioavailability, prevents nicotine-induced vascular dysfunction in rats. It remained unknown whether this effect also occurs in humans. *Methods*. Therefore, 20 healthy volunteers (mean age: 38 years) were investigated twice, 7–10 days apart in a randomized, cross-over, and investigator-blinded design. Vascular function was assessed by flow-mediated vasodilatation (FMD) of the brachial artery and by measurements of the soluble vascular cell adhesion molecule (sVCAM)-1. Investigations were performed after an overnight fast as well as 20 minutes after one cigarette smoking. On another day, the same procedure was applied following a 3-day oral therapy with benfotiamine (1050 mg/day). Ten patients were randomized to start with smoking alone, and ten started with benfotiamine. *Results*. Results are expressed as (mean ± SEM). Smoking acutely induced a decrease in FMD by 50% (^**∗∗**^ *P* < 0.001 versus baseline) an effect significantly reduced by benfotiamine treatment to 25%^**∗**§^ (^**∗**^ *P* < 0.05 versus baseline, ^§^ *P* < 0.05 versus smoking alone). Smoking-induced elevation in sVCAM-1 was also prevented by benfotiamine. The endothelium-independent vasodilatation remained unaltered between days. *Conclusion*. In healthy volunteers, smoking blunts vascular function mirrored by a decrease in FMD and an increase in sVCAM-1. Short-term treatment with benfotiamine significantly reduces these effects, showing protective vascular properties.

## 1. Introduction

Smoking is associated with increased cardiovascular morbidity and mortality [1]. Numerous authors have reported that smoking acutely induces endothelial dysfunction (ED) [2–4]. Repeated, even transient ED episodes are believed to result on long term in persistent ED and atherosclerosis. Indeed, habitual smoking relates in a dose-dependent manner to ED [5]. Mainly 2 pathomechanisms have been incriminated in the pathogenesis of smoking-induced ED: increased oxidative stress (OS) [6] and stimulation of inflammation [7], but other toxic effects of the numerous compounds contained in smoke (over 4000 [8]) are under investigation. Recent data have suggested, for example, that cigarette smoke is a source of advanced glycation end products (AGEs). AGEs have been incriminated in the pathogenesis of cardiovascular as well as diabetes complications, and aging [9].

Improvement of endothelial function was postulated to prevent atherosclerosis [10], and ED induced by smoking is reversed by smoking cessation [5, 11]. Though, there is only a partial recovery of ED following abstinence and rates of smoking cessation are low [12]. Therefore it is important to identify therapeutic approaches that minimize the deleterious effects of smoking on endothelial function.

Several attempts have been made to improve ED in smokers, like treatment with vitamin C [2] and E [3], L-arginine [13], tetrahydrobiopterin, sildenafil [4], folic acid, and other substances [14]. The effects of benfotiamine on endothelial function in smokers have not been investigated yet. Benfotiamine is a prodrug of vitamin B_1_ with much higher bioavailability than thiamine [15] and is commonly used in the treatment of diabetic neuropathy. As a transketolase activator, it directs glucose substrates to the pentose phosphate pathway. Thus, it blocks several hyperglycemia-induced pathways, one of them being endogenous AGE and dicarbonyls formation [16]. But benfotiamine has also antioxidant properties [17, 18], protects endothelial cells under conditions of hyperglycemia [19] in vitro and in animal models, modulates the activity of the nitric oxide synthase [20, 21] (NOS, a critical enzyme that promotes nitric oxide generation), has anti-inflammatory effects [22], and protects against smoke induced endothelial dysfunction in rats [23]. Moreover, we and others [24] have previously shown protective effects of benfotiamine or thiamine on endothelial cells in humans postprandially or during hyperglycemia [25]. Recently, a study has demonstrated in rats that benfotiamine counteracts endothelial dysfunction induced by nicotine [23]. It remained unknown whether this effect occurs in humans too. 

The aim of our study was therefore to investigate the effects of smoking one cigarette on vascular function in 20 otherwise healthy habitual smokers, with and without benfotiamine pretreatment. This study was meant to represent a proof-of-principle for demonstrating protective vascular effects of thiamine's prodrug benfotiamine under nonhyperglycemic conditions using smoking as a common vascular noxae.

## 2. Subjects and Methods

Twenty healthy subjects (age: 38, 18–58 years (mean, range); weight: 72 ± 3 kg, height: 172 ± 2 cm, BMI: 24 ± 1 kg/m^2^, pack-years: 18.3 ± 12.1 (mean ± SEM); male/female: 6/14, (number)) were recruited from the staff of the University Hospital of the Heart and Diabetes Center North Rhine-Westphalia where the study was conducted. 

Endothelial function was assessed by high-resolution ultrasound measurement of the flow-mediated vasodilatation (FMD) of the brachial artery and by assessing soluble vascular cell adhesion molecule (sVCAM)-1 as a serum marker of endothelial function. 

Subjects were studied after giving written informed consent. The local ethics committee approved the study which was carried out according to the principles outlined in the Declaration of Helsinki.

### 2.1. Study Design

Each subject was studied on 2 occasions following an overnight fast. All medications (if any) were withdrawn for at least 12 hours. Each subject was investigated twice, 7–10 days apart in a randomized, cross-over, and investigator-blinded design. On one day (S), vascular function was measured after an overnight fast and 20 minutes after smoking one standard cigarette (10 mg tar, 0.9 mg nicotine). On another day (S + BT), the same procedure was applied following a 3-day therapy with benfotiamine (Milgamma, Woerwag, Germany) given orally on day 1, 2 (3 × 350 mg/d), and 3 (1050 mg one hour prior to smoking). In a randomized fashion, ten patients (50%) started with “S” and continued with “S + BT” after 7–10 days. Ten patients started with benfotiamine pretreatment and “S + BT” and were allowed a wash-out period of 7–10 days before “S.”

Subjects refrained from smoking for at least 12 hours before each test; after test completion and during the wash-out period of 7 days, smoking was permitted ad libitum. On each test day, vascular function was assessed in the fasting state (around 07:00 AM) as well as 20 min following smoking one standard cigarette. Venous blood was drawn after each vascular function measurement. Between tests, volunteers were allowed to stand up and walk but prevented from participating in any major physical activity, drinking, or eating. 

### 2.2. Flow-Mediated Dilatation (FMD) Measurements of Macrovascular Function

Several techniques for the noninvasive assessment of endothelial function are available, with the ultrasound measurement of FMD of the brachial artery being the most frequently used noninvasive technique [26]. Moreover, FMD has been shown to sensitively assess endothelial dysfunction in smokers [27]. The method of measuring FMD has been described in detail elsewhere [25] and was assessed at the right brachial artery (using a protocol described by Celermajer et al. [5]), by measuring the arterial diameter response to reactive hyperemia causing endothelium-dependent dilatation. Measurements of arterial diameter were performed with a high-resolution, two-dimensional ultrasound imaging system ATL HDI 5000 (Advanced Technology Laboratories, Bothell, USA) using B-mode, ECG-triggered ultrasound images obtained with a 12 to 15 MHz linear-array transducer.

Studies were performed at 22–24°C in a dark, quiet room. The study subject rested for at least 10 minutes prior to the first scan and remained in a recumbent position throughout the investigation. A pneumatic tourniquet placed on the forearm of the subject was then inflated at 250 mm Hg for 4.5 minutes. Sixty seconds after cuff deflation, a second scan was recorded for 15 seconds for later measurement of reactive dilatation, in accordance with current guidelines [28]. Subjects were then asked to smoke one standard cigarette within 5 minutes. After smoking, further 20 minutes were allowed for smoking effects to develop and then a new FMD measurement was performed. 

After the FMD measurement, sublingual glycerotrinitrate (GTN) spray (0.4 mg) was administered and 5 minutes later the last data acquisition was made. 

Endothelium-dependent dilatation was defined as the percent change in arterial diameter following reactive hyperemia compared to the baseline diameter (flow-mediated dilatation (FMD)). The endothelium-independent dilatation was calculated as the percent increase in arterial diameter 5 minutes following GTN compared to baseline (and termed GTN).

For each scan (15 seconds), the moment of maximal dilatation was identified and at least three cardiac cycles were analyzed at the end of the diastole. Arterial diameter was automatically measured using appropriate software (HDI Lab, ATL Ultrasound v.1.91) and then averaged between cardiac cycles. The same procedure was applied to the measurements of endothelium-independent vasodilatation. 

B-mode sequences were encoded before storage, and the evaluation was performed for all patients at the end of the study by an investigator (SN) blinded to the clinical parameters of the patient and the study sequence.

 FMD in female was shown to vary during the menstrual cycle, with a decrease during the menstrual phase and comparable values during the luteal and the follicular phases [29]. Therefore, the investigation of female subjects in our study was not performed during the menstrual phase.

### 2.3. Blood Sample Collection and Biochemical Measurements

Blood drawing closely followed each measurement of FMD in the contra lateral arm, and stasis was avoided if possible.

Serum was obtained after centrifugation at 1500 g for 20 minutes at 4°C. Aliquots of 750 *μ*L were stored at −80°C. Serum concentrations of VCAM-1 and thiobarbituric acid reactive substances (TBARSs) were determined using commercially available assays (R&D Systems, Wiesbaden, Germany, and Alexis Biochemicals, Gruenberg, Switzerland, resp.). 

The sVCAM-1 has been proposed to closely mirror endothelial dysfunction, to increase cardiovascular risk, and to predict an increased risk for subsequent cardiovascular events in patients with acute coronary syndrome [30]. This qualifies sVCAM as a marker that reflects not only long-term ED but also short-term changes in endothelial function [25]. TBARSs have been proposed as a marker of oxidative stress [31].

### 2.4. Statistical Analysis

Data were analyzed using SPSS for Windows 12.0. Analyses were performed only in subjects with complete data sets (*n* = 20 for FMD, *n* = 18 for sVCAM, and *n* = 17 for TBARS). Continuous variables are expressed as mean ± SEM. The Shapiro-Wilk algorithm was used to determine whether each variable had a normal distribution. A paired, 2-tailed Student's *t*-test was used to compare the effects of smoking on FMD and serum variables. To assess the effect of pretreatment with benfotiamine, the change (arithmetical difference) in each parameter after smoking versus before smoking was compared (Student's *t* test) between S and S + BT. The level of significance was set at 0.05.

## 3. Results

### 3.1. Effects on FMD and GTN

**Figure 1:**
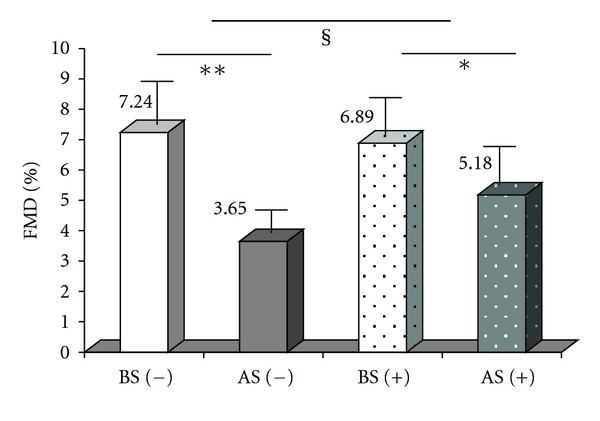
FMD without benfotiamine pretreatment: before smoking (BS (−)) and after smoking (AS(−)), and with benfotiamine pretreatment: before smoking (BS (+)), and after smoking (AS(+)), (**P < 0.001 versus baseline, *P < 0.05 versus baseline, ^§^ *P* < 0.05 with versus without benfotiamine).

Smoking alone induced a significant decrease in FMD of −50%**, an effect that was significantly reduced by pretreatment with benfotiamine to –25%^∗§^ (**P < 0.001, *P < 0.05 after smoking versus before smoking, ^§^ *P* < 0.05 “S + BT” versus “S”) (Figure 1). No significant change in arterial diameter before ischemia occurred following smoking, while the maximal arterial diameter following ischemia was significantly reduced after smoking on both occasions (S and S + BT) (Table 1).

Endothelium-independent vasodilatation (GTN) remained unchanged after smoking on both occasions: 25.7 ± 1.9% (S) and 25.6 ± 2.0% (S + BT).

### 3.2. Effects on Serum Markers of Endothelial Dysfunction

A small, but significant increase in sVCAM followed smoking, an effect completely prevented by pretreatment with benfotiamine (Table 1).

### 3.3. Effects on Oxidative Stress

Following smoking with or without benfotiamine, we found no significant increase in TBARS (Table 1). 

### 3.4. Blood Pressure and Pulse

Values of systolic and diastolic blood pressure (BP) as well as heart rate are depicted in Table 1. Overall, smoking induced a slight, but significant increase in heart rate and systolic and diastolic blood pressure.

## 4. Discussion

The novelty of our study consists in showing that, in chronic, healthy smokers, pretreatment with benfotiamine for 3 days reduces vascular dysfunction induced by smoking one cigarette. In our study, smoking induced a modest, but significant increase in heart rate, systolic and diastolic blood pressure, results in line with previously published data [32].

### 4.1. Data on Macrovascular Function Measured by FMD

As to the mechanisms of benfotiamine and thiamine protecting endothelial cells, it has been demonstrated in cultured endothelial progenitor cells that benfotiamine restores the expression of endothelial NOS decreased by hyperglycemia [19]. In animal models, thiamine deficiency decreases total NOS activity in vulnerable brain regions [21]. These data suggest that NOS activity is sensitive to thiamine concentrations and benfotiamine supplementation prevents the decrease in NOS activity under toxic conditions like hyperglycemia. Since the majority of in vitro studies investigating the protective effects of thiamine/benfotiamine on vascular endothelial cells were performed under hyperglycemic conditions, the exact mechanisms that contribute to endothelial protection from injuries other than hyperglycemia remain to be elucidated. 

An FMD impairment can occur due to three different mechanisms: (1) a decrease in endothelial NO synthesis (endothelial dysfunction), (2) an increase in NO scavenging (e.g., by AGEs, dicarbonyls, or reactive oxygen species- ROS), or (3) a change in NO sensitivity of the smooth muscle cells. Knowing this, we believe that in our study benfotiamine reduced the FMD decrease induced by smoking by exerting positive effects on endothelial function and thus increasing NO bioavailability. What supports this assumption? First, of all we can state that benfotiamine had no effect on the NO sensitivity of the smooth muscle cells (mechanism 3), since the GTN was similar on the 2 study days. Without benfotiamine treatment, smoking induced a small, but significant increase in sVCAM, a marker of endothelial dysfunction, while benfotiamine pretreatment prevented from this increase. An increased scavenging of NO following smoking (mechanism 2) has been demonstrated by previous studies [33]. Whether benfotiamine had any effect on NO scavenging cannot be extrapolated from our data, but it is important to note that no differences in TBARS was noted between days; therefore, no major impact on oxidative stress (OS) is suggested. Our data are consistent with previously published data, which suggested that passive smoking increases markers of OS (8-isoprostane) in nonsmokers but not in smokers [6]. The explanation could be that smokers have an overall increased OS at baseline that masks small changes and that they develop counter-regulatory mechanisms that partly compensate acute OS increases. Indeed, our subjects were young, chronic smokers, and without any known pathology. 

In this study, pretreatment with benfotiamine for 3 days reduced the FMD decrease after smoking by 50% and abolished the increase in soluble markers of endothelial dysfunction (sVCAM-1). Thiamine (vitamin B1) has attracted much attention lately. It has been suggested that thiamine and its prodrug benfotiamine are able to prevent the development of diabetes complications by blocking several hyperglycemia-triggered pathomechanisms and by reducing oxidative stress [16, 18, 34]. Both thiamine and benfotiamine have been shown to prevent hyperglycemia-induced endothelial dysfunction in vitro [35, 36]. In a previous study, we have demonstrated that benfotiamine reduces postprandial endothelial [25] and adipocyte [37] dysfunction in the postprandial state in patients with diabetes mellitus. Another study confirmed that thiamine counteracts endothelial dysfunction under hyperglycemic conditions in subjects with diabetes impaired glucose tolerance, and healthy subjects [24]. These last 2 studies confirm in humans that vitamin B1 protects the endothelium against hyperglycemia-induced damages. The question remained whether this protection is restricted to the hyperglycemic state. Recently, Balacumar et al. suggested that benfotiamine attenuates nicotine and uric acid-induced vascular endothelial dysfunction in the rat [23]. Our present study demonstrates in humans the protective effect of benfotiamine on smoking-induced endothelial dysfunction, adds to the evidence of vasculoprotective effects of benfotiamine, and suggests that benfotiamine protects the endothelium not only against hyperglycemia, but also against injuries under normoglycemic conditions. As to complete the picture of cardiovascular effects of thiamine, it should be mentioned that thiamine was suggested to prevent the development of diabetic cardiomyopathy in animal models [38] and to improve left ventricular ejection fraction in patients with moderate-to-severe congestive heart failure [39].

The potential role of thiamine supplementation might further gain importance in the light of data suggesting that thiamine deficiency is underdiagnosed [40] and often presents in high-risk populations, like people with diabetes mellitus [40] or heart failure [39]. Interestingly, chronic diuretic therapy increases thiamine elimination, potentially contributing to the onset/exacerbation of thiamine deficiency [39]. In smokers, deficient thiamine intake has been reported too [41]. 

In conclusion, our data show that short-term benfotiamine treatment can partly restore macrovascular function in healthy smokers. We consider that the best protection against smoking-induced changes is smoking cessation. Though, our study might contribute to understanding the pathomechanisms that contribute to vascular damage in smokers and help developing strategies for long-term vascular protection in long-term smokers after smoking cessation. It adds to the evidence of beneficial effects of thiamine on endothelial function not only under hyperglycemic conditions, but also in the presence of other noxae and advocates further research on thiamine as an agent with protective cardiovascular potential.

## 5. Limitations of the Study

Our study has several limitations. First, it was not placebo-controlled. Though, acute (−40%) [4] (−70%) [42], 3 days (−50%) [13], and prolonged (4 weeks) (−60%) [3] placebo administration could not prevent FMD decrease induced by acute smoking in different studies (values presented as percent decrease in FMD after smoking compared to baseline under placebo therapy), and the degree of FMD alteration was comparable to that seen during our control day (−50%). Therefore, we do not consider that a placebo effect masked the benfotiamine effect. All persons involved in parameter evaluation (FMD or laboratory) were blinded.

Menstrual cycle and hormonal contraception were shown to influence FMD [29] and sVCAM [43]. To avoid this, female participants were investigated timely apart from the menstrual phase and, due to the randomized, cross-over design, half of the female subjects started with benfotiamine, and half started with placebo, therefore harmonizing the potential influences of the menstrual cycle within the group. As a result, there was a high reproducibility of baseline FMD and sVCAM on the 2 study days, with a day-to-day variation of less than 5% of the initial value.

In a previous study, Papamichael et al. [44] suggested that the maximum impairment in FMD occurs 15–30 minutes following smoking one cigarette and that this effect lasts up to 60 minutes. For this pilot, proof-of-principle study, we decided to perform measurements only at the assumed time of maximum impairment of vascular function (20 minutes). Whether benfotiamine exerts longer-lasting effects on endothelial function after smoking warrants further research. 

The changes in FMD were examined after a 3-day therapy with benfotiamine, and therefore it offers no evidence about the long-term use of this medication.

## Figures and Tables

**Table 1:** Clinical parameters, arterial diameter of the brachial artery and laboratory parameters during the 2 study days.

	Without benfotiamine		With benfotiamine	
	Before smoking	After smoking	Before smoking	After smoking
Systolic blood pressure (mmHg)	121 ± 3	124 ± 3	117 ± 2	121 ± 2*
Diastolic blood pressure (mmHg)	75 ± 2	78 ± 2*	75 ± 2	77 ± 2
Heart rate (beats/min)	69 ± 2	75 ± 2*	68 ± 2	74 ± 2*
Brachial artery diameter at baseline (mm)	3.39 ± 0.95	3.41 ± 0.10	3.52 ± 0.10	3.50 ± 0.12
Postischemic diameter (mm)	3.64 ± 0.09	3.54 ± 0.09*	3.76 ± 0.09	3.67 ± 0.11*
sVCAM (ng/mL)	468 ± 24	488 ± 25*	484 ± 21	484 ± 24
TBARS (nmol/mL)	7.4 ± 0.3	7.5 ± 0.3	7.4 ± 0.5	7.8 ± 0.5

*P < 0.05 after smoking versus before smoking.
